# Psychiatric disorders in term-born children with marginally low birth weight: a population-based study

**DOI:** 10.1186/s13034-024-00714-2

**Published:** 2024-02-08

**Authors:** Shu-I Wu, Yu-Hsin Huang, Kai-Liang Kao, Yu-Wen Lin, Po-Li Tsai, Nan-Chang Chiu, Ching-Hu Chung, Chie-Pein Chen

**Affiliations:** 1 Department of Medicine, MacKay Medical College, #46, Sec. 3, Zhongzheng Rd, Sanzhi Dist., New Taipei City, 252 Taiwan; 2 Department of Psychiatry, MacKay Memorial Hospital, Taipei, Taiwan; 3 Department of Pediatrics, Far Eastern Memorial Hospital, Taipei, Taiwan; 4 School of Nursing, College of Nursing, Taipei Medical University, Taipei, Taiwan; 5 Division of Colorectum, Department of Surgery, MacKay Memorial Hospital, Taipei, Taiwan; 6 Department of Pediatrics, MacKay Children’s Hospital, Taipei, Taiwan; 7 Division of High Risk Pregnancy, MacKay Memorial Hospital, 92 Sec. 2 Zhong-Shan North Road, 104 Taipei, Taiwan

**Keywords:** Marginally low birth weight, Psychiatric disorder, ADHD, Autism, Emotional disturbance

## Abstract

**Background:**

Marginally low birth weight (MLBW) is defined as a birth weight of 2000 ~ 2499 g. Inconsistent findings have been reported on whether children with low birth weight had higher rates of neurological, attention, or cognitive symptoms. No studies have explored the occurrence of clinically diagnosed psychiatric disorders in term- born MLBW infants. We aimed to investigate the risk of subsequent psychiatric disorders in term-born children with MLBW.

**Methods:**

This is a nationwide retrospective cohort study, by analysing the data from Taiwan’s National Health Insurance Research Database from 2008 to 2018. The study population includes propensity-score-matched term-born infants with MLBW and those without MLBW (birth weight ≥ 2500 g). Cox proportional hazard analysis was used after adjustment for potential demographic and perinatal comorbidity confounders. Incidence rates and hazard ratios (HR) of 11 psychiatric clinical diagnoses were evaluated.

**Results:**

A total of 53,276 term-born MLBW infants and 1,323,930 term-born infants without MLBW were included in the study. After propensity score matching for demographic variables and perinatal comorbidities, we determined that the term-born MLBW infants (n = 50,060) were more likely to have attention deficit and hyperactivity disorder (HR = 1.26, 95% confidence interval (CI) [1.20, 1.33]), autism spectrum disorder (HR = 1.26, 95% CI [1.14, 1.40]), conduct disorder (HR = 1.25, 95% CI [1.03, 1.51]), emotional disturbance (HR: = 1.13, 95% CI [1.02, 1.26]), or specific developmental delays (HR = 1.38, 95% CI [1.33, 1.43]) than term-born infants without MLBW (n = 50,060).

**Conclusion:**

MLBW was significantly associated with the risk of subsequent psychiatric disorder development among term-born infants. The study findings demonstrate that further attention to mental health and neurodevelopment issues may be necessary in term-born children with MLBW. However, possibilities of misclassification in exposures or outcomes, and risks of residual and unmeasured confounding should be concerned when interpreting our data.

## Introduction

Low birth weight (LBW) is defined as a birth weight of 1500 ~ 2499 g [1], and small for gestational age (SGA) refers to newborns whose birth weight is less than the 10th percentile for their gestational age [2, 3]. Newborns with LBW or SGA had more than 10 times the risk of perinatal mortality compared with those with a birth weight appropriate for gestational age (AGA) [4]. While survival rates in newborns of LBW or SGA increased with the improvements in obstetric and neonatal care, the rates of neurologic impairment and disability are also likely to increase. Studies have reported that compared with AGA children, SGA children had a three times higher rate of neurological, motor, or learning difficulties, as well as reduced intelligence or attention [5–9]. However, a cohort study that investigated cognitive problems in term-born school-aged children observed no differences between LBW infants and the control group (birth weight 3000 ~ 3499 g), except for coordination and selective attention [10].

Inconsistencies in the aforementioned study findings regarding LBW or SGA infants [11, 12] were likely due to the various screening questionnaires or parental surveys used at different assessment ages [9, 13]. There have already been many studies on neurodevelopmental or behavioral outcomes among low or very low birth weight (VLBW), and most of them were among preterm babies [11, 12]. Nevertheless, the etiology, clinical implications, or prognoses of subsequent psychiatric disorders may be different in term-born marginally low birth weight (MLBW: 2000 ~ 2499 g) [5, 11, 12]. Main outcomes from most previous literature were on behavioral symptoms, learning performances, or internalizing or externalizing disorders obtained by self-report or questionnaires assessments. Large-scale follow-up studies on long-term incidence of subsequent psychiatric disorders diagnosed by clinicians in term-born children with MLBW are scarce [9, 13]. LBW infants (< 2500 g) is a heterogeneous group that includes SGA, preterm, or term infants. Prematurity has been known to be implicated in adverse neurodevelopmental outcomes [14, 15]. Neurodevelopmental or psychiatric outcomes in children with LBW may be related to insufficient gestational weeks from premature birth, or underdevelopment manifested by LBW. Although term-children born with MLBW are not as at-risk for health complications as those born with VLBW or preterm, they may still be at increased risk for a range of health problems, including developmental delays, cognitive impairments, and psychiatric disorders. Studies on term-born babies can avoid the confounding effect of gestational age on neurodevelopment of MLBW. However, no population-based cohort studies to date have been conducted on the occurrence of clinician- verified psychiatric disorders in the less-studied term-born MLBW babies [8, 9, 13]. Addressing these risks early on could have important implications for a child’s long- term health and well-being.

Accordingly, we conducted this 11 year cohort study using a nationally representative population-based dataset in Taiwan to compare the incidence of childhood neurodevelopmental or psychiatric disorders between defined groups of term-born children with MLBW and without MLBW (birth weight ≥ 2500 g) children. We hypothesized that term-born infants with MLBW would have an increased risk of psychiatric disorders that may require public health and clinical intervention.

## Methods

### Data source

On March 1, 1995, the National Health Insurance (NHI) program was established and achieved coverage exceeding 99% of the residents in Taiwan [16]. Each year, the National Health Insurance Administration (NHIA) provides encrypted personal identification, diagnoses, and health-care utilization data in the Taiwan National Health Insurance Research Database (NHIRD). Diseases in the NHIRD are coded using the *International Classifications of Disease, Ninth Revision, Clinical Modification* (*ICD-9-CM*) codes. Because the NHIRD contains only deidentified secondary data, the need for informed consent was waived [16]. In this study, we used the Longitudinal Health Insurance Database (LHID), a subset of the NHIRD [17]. Studies have reported no significant difference in the distribution of age, gender, or health-care costs between the LHID and the general population in Taiwan [18, 19]. The LHID has information on all diagnoses and health-care utilization, including outpatient clinic visits, hospitalizations, and emergency department visits from 2008 to 2018. Furthermore, 96.4% of the total population in Taiwan is the Han Chinese [20]. The ethnic effect on the present results is minimal.

### Patients

We identified newborns with birth weight between 2000 to 2499 g from 2008 to 2015 and assigned them to our MLBW cohort. Those who were diagnosed as having preterm birth (gestational age < 37 weeks) disorders related to short gestation (*ICD-9-CM* 765.xx), had a birth weight of < 2000 g, or died were excluded. Only term-born (gestational age > 37 weeks) singletons were included. Information on the diagnoses and health-care utilization of patients with incident psychiatric disorders was traced until 2018. Information on gestational weeks, Apgar scores, and birth weights for all live births were further obtained from the Taiwan Birth Certificate Registration. In Taiwan, standard practice for determining gestational age entails evaluating the difference between the date of the last menstrual period and the delivery date. Prenatal ultrasonography was used when obstetrical dating was unavailable. For our comparison cohort, we randomly selected term-born infants who had a birth weight of ≥ 2500 g (without MLBW), according to local standards [21], and did not receive a diagnosis of preterm birth (*ICD-9-CM* 765.xx) or LBW (*ICD-9-CM* 764.xx) for the period from 2008 to 2015 from the LHID. Propensity scores were calculated by performing logistic regression on all observed covariates. Term-born infants with or without MLBW that had similar propensity scores were matched at a 1:1 ratio. The calculation of propensity scores by logistic regression was conducted using the SAS 9.4 package. To evaluate the quality of matching, the standardized mean difference (SMD) was set at the value of less than 0.2, and the matching caliper was 0.2.

### Main outcomes

Our primary outcome variables were incident psychiatric disorders such as schizophrenia or schizoaffective disorders (*ICD-9-CM* 295.xx), affective disorders (*ICD-9-CM* 296.xx), psychosis (*ICD-9-CM* 298.xx), adjustment disorders, anxiety (*ICD-9-CM* 300.00), emotional disturbances or oppositional defiant disorders (*ICD-9-CM* 313.xx), conduct disorders (*ICD-9-CM* 312.xx), attention deficit or hyperkinetic syndromes (*ICD-9-CM* 314.xx), personality disorders (*ICD-9-CM* 301), autism spectrum disorders (*ICD-9-CM* 299.0), and developmental delays (*ICD-9-CM* 315.xx) or intellectual disability (*ICD-9-CM* 317–319) identified through linkages with the LHID. In Taiwan, diagnoses of psychiatric disorders were made by doctors according to criteria from Diagnostic and Statistics Manual- IV-TR [22] and DSM5 [23]. Clinical psychiatric diagnoses given by psychiatrists, pediatricians, or physicians for at least one inpatient or three outpatient records were identified from the NHIRD [24] as our outcomes of interest. Selections of these diagnoses as our outcomes were based on past literature exploring risks of psychiatric disorders, externalizing or internalizing disorders, cognitive disorders, or behavioral problems in children born preterm or VLBW [25, 26]. Follow-up time was calculated in person-years from the index date (date of birth) until the date of first diagnosis of psychiatric disorder from at least three outpatient visits or one hospitalization to ensure the validity of the diagnoses, the end of 2018, or withdrawal from the insurance system due to death or loss to follow-up [27].

### Covariates

The following covariates were included in the process of propensity score matching and Cox regression analysis: sociodemographic data (including sex; age on December 31, 2015; and family income level) and severe neonatal morbidities (such as hemorrhage, grade III–IV intraventricular hemorrhage [28], cystic periventricular leukomalacia [29], periventricular leukomalacia, hydrocephalus, neonatal seizures, severe retinopathy of prematurity stages 4 or 5, respiratory distress syndrome, pneumonia, bronchopulmonary dysplasia, necrotizing enterocolitis [30], maternal–fetal infection, sepsis, resuscitation at birth, or a stay in a neonatal intensive care unit). All neonates with congenital malformations or lethal chromosome anomalies such as trisomy 13 and 18 were excluded. All diagnoses used were obtained from the NHIRD. The NHIA regularly conducts expert reviews of all disease diagnoses and treatments to ensure accuracy. Previous research has confirmed a moderate to substantial level of agreement (Kappa 0.55–0.86) between diagnoses in the NHIRD and the gold standard of medical records [31, 32]. This retrospective cohort study was approved by the MacKay Memorial Hospital Institutional Review Board (MMHIRB; 17MMHIS114).

### Statistical analysis

The chi-square test (or Fisher’s exact test) and t test were used to identify differences between the MLBW and comparison cohorts in terms of categorical and continuous variables, respectively. A *p* value of > 0.05 for differences between the study cohort and propensity-score-matched comparison cohort was considered to represent sufficiently matched cohorts. Univariate and multivariable Cox proportional hazard regression analyses were performed using the SAS 9.4 package (SAS Institute Inc., Cary, NC, USA) to compare the incidence of individual and overall psychiatric disorders between the two cohorts.

## Results

**Fig. 1:**
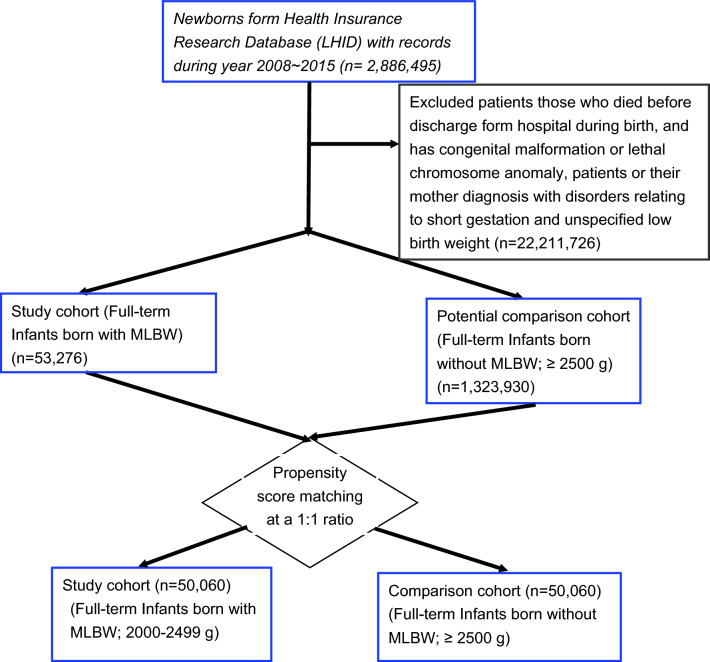
Flow Chart of Selection of Study and Comparison Subjects from Patients registered in Taiwan’s Longitudinal Health Insurance Research Database (LHID), a subset of the Taiwan’s National Health Insurance Research Database. Propensity score matching by: the family’s levels of income and neonatal data (including sex, birth weight, blindness and low vision, infections specified to the perinatal period, necrotizing enterocolitis in fetus or newborn, epilepsy, fetal and neonatal hemorrhage, communicating hydrocephalus, intrauterine hypoxia and birth asphyxia, other perinatal jaundice, microcephalus, patent ductus arteriosus, pneumonia, pneumothorax, respiratory distress syndrome, other respiratory conditions of fetus and newborn). *MLBW* marginally low birth weight, defined as birth weight between 2000 and 2499 g

From a total of 2,886,295 newborns registered in the LHID between 2008 ~ 2015, we identified our study subjects of 53,276 term-born infants with MLBW. Figure 1 illustrates the selection and matching criteria for our study and comparison cohorts. There were 1,323,930 enrollees left as potential comparison subjects (term-born infants without MLBW, Fig. 1, Table 1) after excluding patients with congenital malformation, chromosome anomaly, or mothers diagnosed with disorders relating to short gestation and LBW (n = 22,211, Fig. 1). As presented in Table 1, all demographic characteristics and perinatal comorbidities (except for pneumothorax, blindness, and low vision) were significantly different between the term-born infants with or without MLBW before propensity score matching. A higher proportion of the MLBW infants had epilepsy, fetal and neonatal hemorrhage, communicating hydrocephalus, intrauterine hypoxia and birth asphyxia, perinatal jaundice, microcephalus, patent ductus arteriosus, pneumonia, and other respiratory conditions compared with the infants without MLBW (Table 1). Parental histories of psychiatric disorders were also compared and no significant differences were found between children with or without MLBW. We then calculated propensity scores for all participants in Table 1, and selected participants with similar propensity scores from both the MLBW and the non-MLBW groups to match at a 1:1 ratio (Fig. 1). Characteristics of participants after propensity scores matching are shown in Table 2. After propensity score matching for demographic variables and perinatal comorbidities, there were 50,060 term-born infants with MLBW, and 50,060 term-born infants without MLBW. These two cohorts did not differ significantly in terms of demographic status or proportions of comorbidities (Table 2 and Fig. 1).

**Table 1:** Demographic characteristics before matching

Variables	MLBW	No MLBW	*p* value
	*n *= 53,276 (%)	*n* = 1,323,930 (%)	
Sex			< 0.0001^a^
Female	32,080 (60.21)	634,531 (47.93)	
Male	21,196 (39.79)	689,399 (52.07)	
Age^c^			
Mean ± SD	3.27 + 2.31	3.39 + 2.32	<0.0001^b^
Economic status			< 0.0001^a^
Low income	3785 (7.10)	66,293 (5.01)	
Confounders			
Blindness and low vision	3 (0.01)	26 (0.002)	0.0705^a^
Epilepsy	241 (0.45)	3101 (0.23)	< 0.0001^a^
Fetal and neonatal hemorrhage	35 (0.06)	236 (0.02)	< 0.0001^a^
Communicating hydrocephalus	10 (0.02)	83 (0.01)	0.0006^a^
Intrauterine hypoxia and birth asphyxia	38 (0.07)	201 (0.02)	< 0.0001^a^
Other perinatal jaundice	54 (0.10)	714 (0.05)	< 0.0001^a^
Microcephalus	35 (0.07)	123 (0.01)	< 0.0001^a^
Patent ductus arteriosus	459 (0.86)	3069 (0.23)	< 0.0001^a^
Pneumonia	1707 (3.20)	36,133 (2.73)	< 0.0001^a^
Pneumothorax	9 (0.02)	135 (0.01)	0.1383^a^
Other respiratory conditions of fetus and newborn	338 (0.63)	1593 (0.12)	< 0.0001^a^

*MLBW* marginally low birthweight, defined as term-born infants with birth weight between 2000 and 2499 g. No MLBW: term-born infants with birth weight ≥ 2500 g

^a^Chi-squared test

^b^*t*-test

^c^Age was calculated using 2013/12/31 as the time point to minus the date of birth

**Table 2:** Demographic characteristics after propensity score matching

Variables	MLBW (n = 50,060)	No MLBW (n = 50,060)	p value
Gender			>0 .999^a^
Female	30,115 (60.16)	30,115 (60.16)	
Male	19,945 (39.84)	19,945 (39.84)	
Age^c^			>0 .999^b^
Mean ± SD	3.26 + 2.31	3.26 + 2.31	
Economic status			> 0.999^a^
Low income	3543 (7.08)	3543 (7.08)	
Confounders			
Blindness and low vision	3 (0.01)	3 (0.01)	>0 .999^a^
Epilepsy	180 (0.36)	180 (0.36)	>0 .999^a^
Fetal and neonatal hemorrhage	12 (0.02)	12 (0.02)	>0 .999^a^
Communicating hydrocephalus	4 (0.01)	4 (0.01)	>0 .999^a^
Intrauterine hypoxia and birth asphyxia	15 (0.03)	15 (0.03)	>0 .999^a^
Other perinatal jaundice	37 (0.07)	37 (0.07)	>0 .999^a^
Microcephalus	12 (0.02)	12 (0.02)	> 0.999^a^
Patent ductus arteriosus	363 (0.73)	363 (0.73)	>0 .999^a^
Pneumonia	1592 (3.18)	1592 (3.18)	> 0.999^a^
Pneumothorax	3 (0.01)	3 (0.01)	> 0.999^a^
Other respiratory conditions of fetus and newborn	217 (0.43)	217 (0.43)	> 0.999^a^

MLBW: marginally low birthweight, defined as term-born infants with birth weight between 2000 and 2499 g. No MLBW: term-born infants with birth weight ≥ 2500 g

^*a*^Chi-squared test; ^b^ *t*-test

^c^Age was calculated using 2013/12/31 as the time point to minus the date of birth

Table 3 presents the associations between MLBW term-born infants and psychiatric disease outcomes during our follow-up period (2008–2018). After adjustment, our result revealed that the MLBW infants were more likely to have subsequent diagnoses of specific delays in development or intellectual disability (hazard ratio [HR] = 1.38, 95% CI [1.33, 1.43]), attention deficit hyperactivity disorder (ADHD; HR = 1.26, 95% CI [1.20, 1.33]), autism spectrum disorder (ASD; HR = 1.26, 95% CI [1.14, 1.40]), conduct disorder (HR = 1.25, 95%CI [1.03, 1.51]), and emotional disturbance (HR = 1.13, 95% CI [1.02, 1.26]). The HR for adjustment disorders, affective disorders, anxiety disorders, personality disorders, or psychosis did not differ significantly between the two cohorts (Table 3).

**Table 3:** Univariate and multivariate analyses of subsequent psychiatric disease incidences in full term infants with or without the diagnosis of low birth weight (birth weight between 2000 to 2500 g) (N = 100,120)

variable	MLBW (n = 50,060)	No MLBW (n = 50,060)	Univariate model				Multivariate model^a^			
	No. of participants	No. of participants	*Crude HR*	*(95% CI)*^b^		*p value*	*Adjusted HR*	*(95% CI)*^b^		*p value*
Any psychiatric disorders	8660	6695	1.36	1.36	1.40	< 0.001	1.33	1.29	1.37	< 0.001
Specific delay in development or mental retardation	7109	5290	1.40	1.35	1.46	<0 .001	1.38	1.33	1.43	<0 .001
Attention deficit and hyperactivity disorder	3359	2690	1.27	1.20	1.34	<0 .001	1.26	1.20	1.33	< 0.001
Adjustment disorders	240	228	1.05	0.88	1.26	0.58	1.05	0.88	1.26	0.58
Afftve disorders	12	8	1.50	0.61	3.67	0.37	1.50	0.61	3.67	0.37
Anxiety disorders	319	292	1.09	0.93	1.28	0.27	1.09	0.93	1.28	0.27
Autism spectrum disorders	854	677	1.27	1.14	1.40	< 0.001	1.26	1.14	1.40	<0 .001
Conduct disorders	234	188	1.25	1.03	1.51	0.03	1.25	1.03	1.51	0.02
Emotional disturbance	704	622	1.13	1.02	1.26	0.02	1.13	1.02	1.26	0.02
Personality disorders	14	6	2.33	0.90	6.07	0.08	2.33	0.90	6.07	0.08
Psychosis	5	0	1.50	0.25	8.98	0.66	1.50	0.25	8.99	0.66
Schizophrenia	0	0								

*MLBW* marginally low birthweight, defined as term-born infants with birth weight between 2000 and 2499 grams. No MLBW: term-born infants with birth weight 2500 grams

^a^ Multivariate Cox proportional hazard model, adjusted for all covariates from Table 2. ^b^ No MLBW is reference group

## Discussion

This is the first and largest study to use a population-based dataset to examine the impact of MLBW on subsequent psychiatric disorders. The study findings suggest that term-born children with MLBW were more likely than term-born children without MLBW to be diagnosed as having developmental delays or intellectual disability, ADHD, ASD, conduct disorder, and emotional disturbances.

Our finding that term-born infants with MLBW were more likely to have developmental delays supports the findings of previous studies that have reported an increased association of lower intelligence, cognitive difficulties, or lower school achievements in term-born SGA infants compared with those with normal birth weights [2, 6, 33]. The mechanisms underlying developmental delays or intellectual impairment are still unclear but may be a consequence of perinatal brain injuries disrupting cortical growth [34]. Placental insufficiency is another possible factor affecting fetal cerebral hemodynamics leading to deficits in brain development [2, 35]. However, placental insufficiency may be a confounding factor for poor neurodevelopmental outcomes, such as language, intelligence, or cognitive developmental disorders. Other possible causes, including maternal factors such as mothers’ genetic loading, incident prenatal infections, or intrauterine exposure to cigarettes, alcohol, or narcotic drugs and their associations with intellectual disability or developmental disabilities may also be considered [2]. Nonetheless, placental insufficiency may not be associated with neurodevelopmental problems. Savchev et al. reported that although SGA infants had poorer neurological developments, the prenatal umbilical artery Doppler assessment revealed no signs of placental insufficiency [5]. Future investigations are required to examine possible causal factors for developmental issues in term-born infants with MLBW and genetic or intrauterine exposures.

Our finding that term-born infants with MLBW had an increased risk of ADHD is consistent with those of previous studies that reported an increased association of LBW or SGA with attentional problems or aggressive behaviors [8, 9, 36]. Multifactorial interplays of genes and environmental factors are implicated in the causal mechanisms underlying ADHD [37]. Some brain imaging studies have reported that reductions in the brain surface area, cortical gray matter volume, or brain connectivity were associated with placental insufficiency and may be related to poor neurodevelopment and later attentional problems in SGA babies [36, 38–41]. But other studies also mentioned that such phenomenon was not found in term-born adolescents with SGA [8, 42]. To explore possible causal mechanisms for these findings, additional long-term follow-up studies that involve well-defined phenotypes of ADHD and combine structural or functional neuroimaging techniques for term-born children with MLBW are recommended.

The increased risk of ASD observed in our MLBW cohort is consistent with the findings of previous research that has described associations of prematurity [43] and VLBW [44] with the development of ASD. Individuals with ASD are characterized by limited social interactions, deficits in communication or language skills, restricted interests, or stereotypy [45]. Although ASD is considered a neurodevelopmental disease strongly influenced by genetic factors, the exact cause is unclear and may include genetic, environmental, perinatal infections, or immunological effects [45]. Placental insufficiency that leads to LBW may also cause a brain maturation defect, deficiency of neuroprotective factors, or increased inflammation, thus increasing the risk of brain injury or neurodevelopmental disorders [43]. Most studies to date have focused on the associations between ASD and prematurity or VLBW. Investigations into relevant causal mechanisms underlying the association between term-born MLBW and ASD are still warranted.

Emotional disturbance is characterized by withdrawal, introversion, elective mutism, interpersonal relationship problems, academic underachievement, or oppositional defiant disorders. We found that the MLBW cohort had an increased risk of subsequent diagnoses of emotional disturbance and conduct disorder. This finding is supported by reports that emotional and behavioral problems are more common in children born preterm with VLBW than in term-born children [46]. However, studies that included children born at 32 ~ 36 weeks or full term and conducted follow-up evaluations for preschool behavioral and emotional problems have not revealed SGA as a risk for such disorders when compared with a term-born pediatric population [47, 48]. The discrepancy between our findings and those of the aforementioned studies may be that the inclusion criteria for LBW in our study is different from past research. Past research focused on incidences of emotional or behavioral problems among term babies with SGA or those born at 32 ~ 36 weeks, while our study focused on that among term-born marginally LBW infants (2000 to 2499 g). Furthermore, our outcome of emotional disturbance was determined based on clinical diagnoses from insurance claims, while previous literature determined their outcomes through responses from questionnaires [47, 48]. Studies have demonstrated that the prevalence of poor social skills, inadequate emotional regulation, and associated poor clinical outcomes in patients with ADHD or ASD may be as high as 70% [46, 49]. Research on the probable mechanisms of these conditions should consider the cerebral pathology associated with LBW related to impulsive symptoms [50]. Although parental demographic and child-rearing factors such as mother’s education level, family’s problem-solving abilities, child-rearing style, or parental physiological or psychological distress [51–53] may be associated with incident emotional disturbances in MLBW term-born children, it may be of interest why the incidence of personality disorder was not significantly higher. The main reason may be that the diagnosis of personality disorders usually requires long-term observations of behavioral patterns by mental health clinicians. Usually the diagnosis of personality disorder cannot be made until 18 years old because the personality is still developing before age 18 [54]. It is possible that our study subjects were not old enough to be diagnosed with personality disorders during our observation period between 2008 to 2018. Further research is required to confirm the correlations of the etiology of emotional disturbances or conduct disorders with MLBW.

Our finding that term-born infants with MLBW had increased risk of conduct disorder was in line with a previous study describing children and adolescents born moderate to late preterm had relatively more conduct problems than those born full term [55]. Although there is less consensus about the association between birth weight and conduct problems, symptoms of conduct disorder were also shown by other researchers to be lower in children with LBW/preterm birth than those who were born at term [56]. Probable reasons for the discordance from past studies or with our study may be due to variations in participants’ characteristics of sex, level of prematurity, ranges of birth weight, sample sizes, severities, or definitions for study outcomes (eg. clinical or subclinical symptoms, social or financial adversities, or externalizing or internalizing disorders). Underlying explanations for the increased association between MLBW and conduct disorder in our study may be attributed to a combination of factors associated with LBW, such as possible disruptions in brain development or interactions of nature and nurture. For instance, SGA term fetuses with cerebral blood flow redistribution has been related to adverse neurodevelopmental outcome, especially communication and problem-solving at 2 years old [57]. Poor neurodevelopmental outcome may be confounded by placental dysfunction [2]. Diminished volume in the brain region of hippocampal found in very preterm children [58] has been shown to link to poor impulse control, social incompetence, damaged cognition [59], or neuroticism or introversion personality traits [60, 61]. Whether these mechanisms may account for the developmental deficits found in term-born children with MLBW are still obscure. From the understandings with the possible etiology of conduct disorders, other unmeasured confounders by indication, such as intrauterine exposures to parental smoking or substance misuse, maternal depression, social deprivation, or parental psychosocial stressors, might also interact with the nurturing environment or parenting styles of upbringing in early or late childhood [62], and may lead to behavioral problems. Overall, the increased risk of conduct disorder in term-born children with MLBW is likely to be multifactorial, involving complex interactions between biological, psychological, and social factors. Further research is needed to better understand the possible mechanisms and develop effective prevention and intervention strategies.

The use of a large and nationally representative population-based birth cohort with perinatal data allowed determination of risks without recall bias. This was a major strength of this study. The study carefully matched the control group, which enhanced the reliability of our findings. Nevertheless, the study has several limitations. First, the possibility of misclassifying psychiatric outcomes is a potential limitation inherent to population-based studies. Diagnosis may be influenced by multiple factors, eg. healthcare access, knowledge, education, family support, denial, or opportunity. Although these diagnoses were made by clinicians and may reflect actual observations, they were not obtained through psychiatric diagnostic interviews and may not be valid for research purposes [32]. Second, despite our efforts to minimize differences in demographic and comorbidity variables when matching our study and comparison cohorts, there may still be potential confounders. We find there were no significant differences in prevalence of parental psychiatric history between parents of children with or without MLBW, but histories of tobacco use, dietary habits, and psychosocial stressors are not available in the NHIRD and could not be controlled for.

Furthermore, limited by the use of NHIRD database, parental psychiatric history was not used as a covariate in propensity score matching, which might causally affect the outcome although the effect is minimal. However, even with the past divide of nature versus nurture, more recent evidence has revealed the regulating roles of neuronal connectivity and neural plasticity on the control of behavioral changes and higher cognitive functions [63]. Future establishment of a sibling comparison study may provide more insight. Third, although we used a large population-based sample representative of Taiwan’s population, these findings may have limited generalizability to other countries due to differing ethical considerations or national healthcare system. Fourth, despite a cohort design that enabled us to track our study population for 11 years (2008–2018), our follow-up period was not long enough to investigate incidences of mental illnesses emerging after puberty. For instance, some of the psychiatric diagnoses, including schizophrenia or affective disorders, are not commonly diagnosed before the age of 10 and that the cohort is too young to be diagnosed with these. Further long-term follow-up research using psychiatric diagnostic interviews to determine the occurrences of adult psychiatric disorders may help overcome these limitations.

## Conclusion

Our results reveal that compared with those without MLBW, MLBW term-born children may have an increased risk of mental health issues associated with developmental delays or intellectual disability, ADHD, ASD, conduct disorder, or emotional disturbances. Given the magnitude of the associations observed, the possibility of misclassifications in exposure or outcomes, and risks of residual and unmeasured confounding should be concerned when interpreting our data. However, our findings can still augment the knowledge in prenatal and postnatal parental counseling in term-born children with MLBW. Appropriate follow-up on developmental milestones or assessment of the psychosocial environment among the population at risk would enhance early identification of issues and relevant interventions.

## Data Availability

Datasets being analyzed and results being generated and reported in this article are not publically available due to protections of personal privacy. Restrictions applied to these data, which were used under license for our study, and so are not publicly available for duplication. Further data analysis may be requested after discussion with authors.
